# A Novel RNA-Seq-Based Model for Preoperative Prediction of Lymph Node Metastasis in Oral Squamous Cell Carcinoma

**DOI:** 10.1155/2020/4252580

**Published:** 2020-08-31

**Authors:** Bo Qiao, Min Zhao, Jing Wu, Huan Wu, Yiming Zhao, Fanhao Meng, Yu Tian, Situo Wang, Jinlong Shi, Haizhong Zhang

**Affiliations:** ^1^Medical School of Chinese PLA, Beijing 100853, China; ^2^Department of Stomatology, The First Medical Centre, Chinese PLA General Hospital, Beijing 100853, China; ^3^Medical Big Data Centre, Chinese PLA General Hospital, Beijing 100853, China; ^4^Department of Gastroenterology and Hepatology, Hainan Hospital of Chinese PLA General Hospital, Sanya, 572013 Hainan, China

## Abstract

**Objective:**

To develop and validate a novel RNA-seq-based nomogram for preoperative prediction of lymph node metastasis (LNM) for patients with oral squamous cell carcinoma (OSCC).

**Methods:**

RNA-seq data for 276 OSCC patients (including 157 samples with LNM and 119 without LNM) were downloaded from TCGA database. Differential expression analysis was performed between LNM and non-LNM of OSCC. These samples were divided into a training set and a test set by a ratio of 9 : 1 while the relative proportion of the non-LNM and LNM groups was kept balanced within each dataset. Based on clinical features and seven candidate RNAs, we established a prediction model of LNM for OSCC using logistic regression analysis. Tenfold crossvalidation was utilized to examine the accuracy of the nomogram. Decision curve analysis was performed to evaluate the clinical utility of the nomogram.

**Results:**

A total of 139 differentially expressed RNAs were identified between LNM and non-LNM of OSCC. Seven candidate RNAs were screened based on FPKM values, including NEURL1, AL162581.1 (miscRNA), AP002336.2 (lncRNA), CCBE1, CORO6, RDH12, and AC129492.6 (pseudogene). Logistic regression analysis revealed that the clinical N stage (*p* < 0.001) was an important factor to predict LNM. Moreover, three RNAs including RDH12 (*p* value < 0.05), CCBE1 (*p* value < 0.01), and AL162581.1 (*p* value < 0.05) could be predictive biomarkers for LNM in OSCC patients. The average accuracy rate of the model was 0.7661, indicating a good performance of the model.

**Conclusion:**

Our findings constructed an RNA-seq-based nomogram combined with clinicopathology, which could potentially provide clinicians with a useful tool for preoperative prediction of LNM and be tailored for individualized therapy in patients with OSCC.

## 1. Introduction

Oral squamous cell carcinoma (OSCC) accounts for 95% of all oral malignancies, and its five-year survival rate is up to 50%-60% [1]. Lymph node metastasis (LNM) is considered to be an independent prognostic factor of OSCC, which is associated with tumor recurrence and prognosis [2–4]. Only 25-40% of OSCC patients with LNM at diagnosis will survive 5 years, compared to approximately 90% of those without LNM (non-LNM) [5]. Therefore, accurate assessment of the nodal status and decision about concurrent cervical lymph node dissection is of utmost importance for prognosis and therapy of OSCC.

Unfortunately, there is still no widely accepted method for noninvasive detection for preoperative prediction of LNM in OSCC currently. For OSCC patients with clinically negative neck (cN0), whether to perform cervical lymph node dissection remains a hot topic. Using current methods to predict LNM, approximately 70% of patients with cN0 OSCC who undergo elective neck dissection (END) are found to be pathologically node negative [6]. It is imminent to best select patients with LNM who will benefit from END and to decrease the cost and morbidity of neck dissection in those without LNM [7].

Emerging sequencing technologies in genomics and transcriptomics have characterized many types of human cancers in specific molecules, which provide a critical relationship between cell phenotypes and their molecular characteristics, and new biomarkers or therapeutic strategies for patients [8, 9]. To our knowledge, no nomograms based on RNA-seq have been used to predict LNM in OSCC. We hypothesized that RNA-seq-based nomograms could improve the prediction of LNM in OSCC, so that patients who will benefit from END can be more accurately identified, while retaining END is unlikely to be beneficial. In this study, logistic regression analysis was used to screen for high risk factors for OSCC patients with LNM. We aimed to establish and verify a novel RNA-seq-based nomogram combined with clinicopathological factors to predict LNM in OSCC patients, which may provide an auxiliary tool for personalized precise treatment and assist the clinical therapy decision for OSCC patients.

## 2. Materials and Methods

### 2.1. Data Acquirement

OSCC RNA-seq were obtained from The Cancer Genome Atlas database (TCGA) (https://portal.gdc.cancer.gov/). Screening conditions were as follows: (a) primary sites: hard palate, lip, oral cavity, or oral tongue; (b) disease type: squamous cell neoplasms; (c) experimental strategy: RNA-seq; (d) sample type: primary tumor; and (e) clinical information was composed of AJCC pathological N status. Finally, a total of 276 OSCC samples with gene expression data and corresponding clinical information were utilized for this study, including 157 samples with lymph node metastasis (LNM) and 119 samples without LNM (non-LNM).

The GSE9844 microarray dataset was downloaded from the Gene Expression Omnibus (GEO) database (https://www.ncbi.nlm.nih.gov/gds), including 26 oral tongue squamous cell carcinoma (OTSCC) samples [10]. Among them, 11 samples had lymph node metastasis.

### 2.2. Differential Expression Analyses

Differential expression analyses were performed between LNM and non-LNM OSCC samples using the DESeq2 package in R (version 1.18.1) [11]. Differentially expressed RNAs (DERNAs) were identified in line with adjusted *p* value < 0.05 and ∣log2 fold change (FC) | >1. The overall distribution of DERNAs was visualized into the volcano plot. A functionally grouped network of pathways was constructed using the ClueGO (version 2.5.1) [12, 13] of Cytoscape (version 3.6.1) [14]. The “load marker list” was set to differential gene sets for enrichment analysis, the “show only pathways with *p* value” was set to 0.05, and other settings were set to default.

### 2.3. Variable Selection Method

The chi-square (*χ*^2^) test was used to analyze the difference of patient demography, risk exposure, clinical characteristics, and histopathological features between LNM and non-LNM OSCC samples by SPSS software (version 24.0). *p* < 0.001 was considered statistically significant. Characteristic genes related to LNM were screened utilizing the Boruta package in R [15] (version 6.0.0) based on FPKM values from the expression profile of the GSE9844 dataset, which were plotted into a box plot by the ggplot2 package (version 3.2.1).

### 2.4. Classification Model Fitting and Validation

After removing the samples with incomplete clinical N stage (cN) information, a total of 265 samples were retained and randomly divided into the training set and the test set by a ratio of 9 : 1 while the relative proportion of non-LNM and LNM groups was kept balanced within each dataset. A classification model of the 10-fold crossvalidation was constructed utilizing the R language (version 3.4.4). In a training set, a RF model was built using the “random forest” package in R (version 4.6-14) [16], followed by support vector machine (SVM) model by the “e1071” package in R (version 1.7-2) [17]. Basic function “glm” in R (version 3.4.4) was used to fit the generalized logistic regression model, and the “XGBoost” package in R (version 0.90.0.2) [18] was utilized to implement the XGBoost model. The accuracy, sensitivity, and specificity of the four models were evaluated on the test set according to the calibration curve and ROC curve by the “rms” package (version 5.1-3.1) [19] and “pROC” package (version 1.15.3) in R. Area under the curve (AUC) was used to compare the diagnostic performance of the models. Furthermore, the nomogram of the logistic regression model was performed by the “regplot” package in R (version 0.2) [20]. Then, decision curve analysis (DCA) was conducted to estimate the clinical value of our nomogram between two groups using the “rmda” package (version 1.6) [21], which could analyze the net benefit of the cN and RNA scores for prediction of LNM for OSCC patients.

## 3. Results

### 3.1. Clinical Characteristics

**Figure 1:**
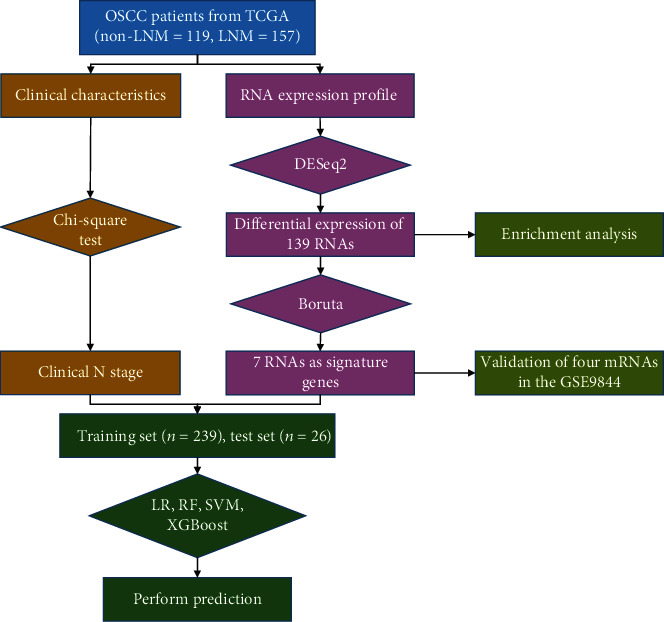
A work flowchart for this study. TCGA: The Cancer Genome Atlas; non-LNM: non lymph node metastasis; LNM: lymph node metastasis; LR: logistic regression; RF: random forest; SVM: support vector machine.

Our study developed a model for the preoperative prediction of LNM.  Figure 1 illustrates the work flowchart of the study. A total of 276 patients with OSCC were included in our study.  Table 1 shows the demographic data and pathological characteristics of these patients. Among them, 157 (56.88%) patients had LNM. 265 patients possessed complete clinical N status; among them, 44 had no lymph node metastasis in clinical examination (cN-) but metastasis occurred in the pathological diagnosis (pN+). 22 patients' clinical examination indicated lymph node metastasis (cN+), but pathological examination results showed no lymph node metastasis (pN-). Furthermore, our data showed that clinical N was significantly different between the two cohorts (*p* < 0.001). However, there were no significant differences between the two groups in terms of neoplasm histologic grade, tobacco smoking history, and anatomic neoplasm subdivision.

### 3.2. Identification of Differentially Expressed RNAs between Non-LNM and LNM of OSCC

**Figure 2:**
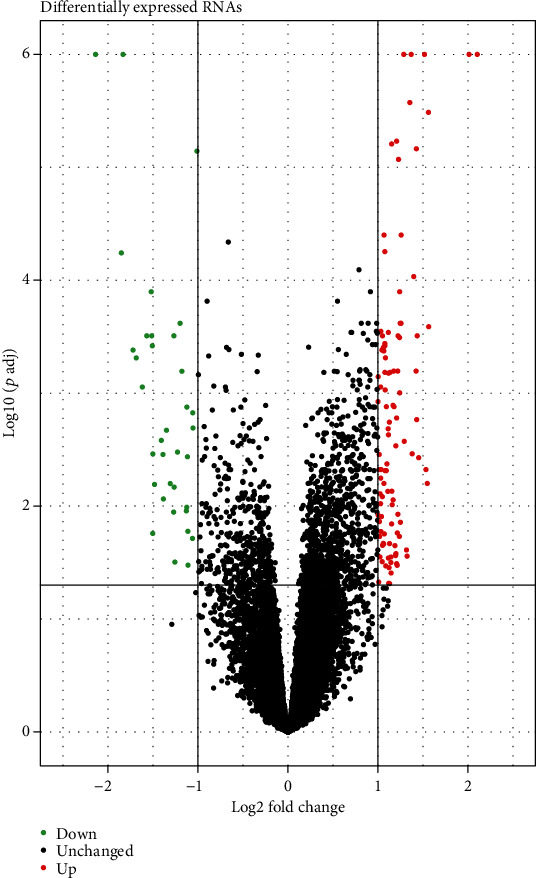
139 differentially expressed RNAs between non-LNM and LNM of OSCC. Red dot represents up-regulation and green dot represents down-regulation.

**Figure 3:**
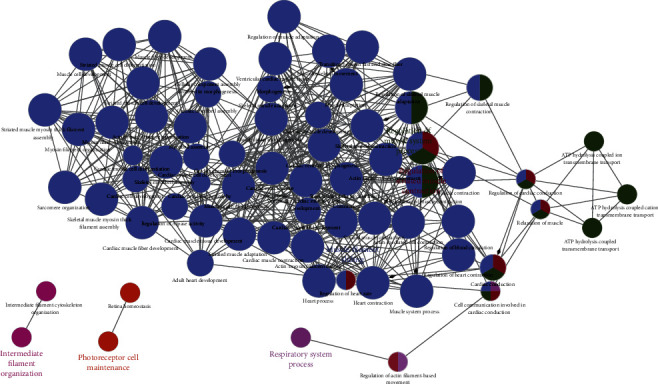
Network of functional enrichment results of differentially expressed RNAs between non-LNM and LNM of OSCC. Each node in the figure represents a term, the connection between the nodes reflects the correlation between the terms, and the color of the node reflects the enrichment classification of the node.

Volcano plots visualized that there were 139 DERNAs between non-LNM and LNM OSCC (Figure 2), including 104 upregulated and 35 downregulated genes. The specific DERNAs are listed in Supplementary Table 1. To analyze the underlying biological function of DERNAs, functional enrichment analysis was carried out by ClueGO and the database called by the GO biological process in ClueGO. Significant biological processes enriched by DERNAs are shown in Figure 3. These DEmRNAs were mainly involved in “regulation of striated muscle contraction,” “regulation of muscle system process,” and “muscle filament sliding.” These results indicated that a variety of biological processes of muscle could be involved in lymph node metastasis of OSCC.

### 3.3. Selection of Candidate DERNAs to Predict LNM of OSCC

**Figure 4:**
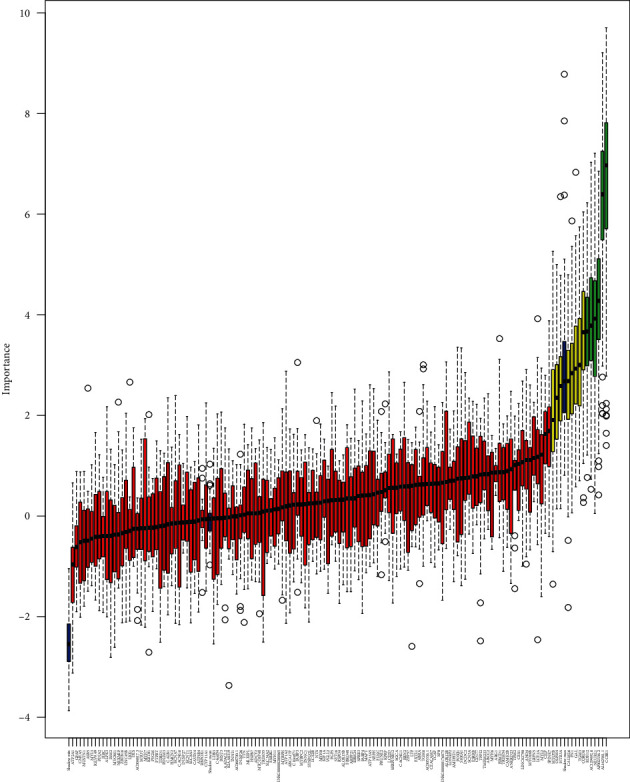
Predictive power of individual gene assessed by Boruta feature selection algorithm. Blue represents the minimum, average and maximum Z-score of the shadow gene. Red and green suggest the Z-Score of the rejection attribute and confirmation genes. Yellow expresses suggestive genes.

**Figure 5:**
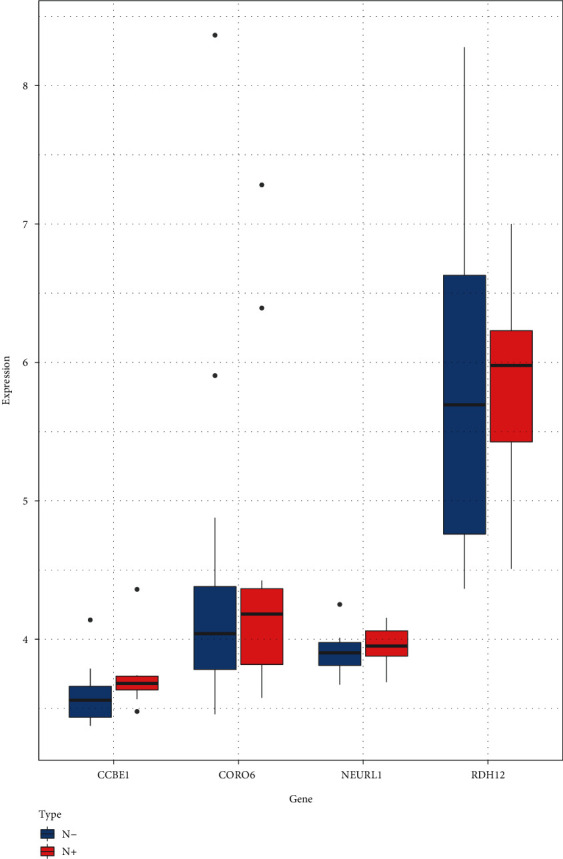
Box plots depicting the expression levels of CCBE1, RDH12, CORO6 and NEURL1 between non-LNM and LNM of OSCC using the GSE9844 dataset. N-:pathological examination without lymph node metastasis. N+: pathological examination with lymph node metastasis.

The Boruta algorithm was used to screen out the signature genes to distinguish non-LNM and LNM of OSCC.  Figure 4 shows the change in *Z*-score of 139 DERNAs during Boruta's operation. The blue boxplots indicated the minimum, average, and maximum *Z*-scores of the shadow gene. The red and green boxplots indicated the *Z*-score of the rejection attribute and confirmation genes, and yellow colors represented suggestive genes. These findings showed that the most important shadow attribute *Z*-score clearly separated important and nonimportant genes. Finally, a total of seven candidate DERNAs were identified for distinguishing non-LNM and LNM of OSCC, including NEURL1, AL162581.1 (miscRNA), AP002336.2 (lncRNA), CCBE1, CORO6, RDH12, and AC129492.6 (pseudogene). Among them, expression profiles of four DERNAs were obtained from the expression profile of the GSE9844 dataset. In Figure 5, we found that the expression levels of CCBE1, CORO6, NEURL1, and RDH12 were significantly higher in N+ compared to N- OSCC patients.

### 3.4. Development and Validation of a Machine Learning Model to Predict LNM of OSCC

**Figure 6:**
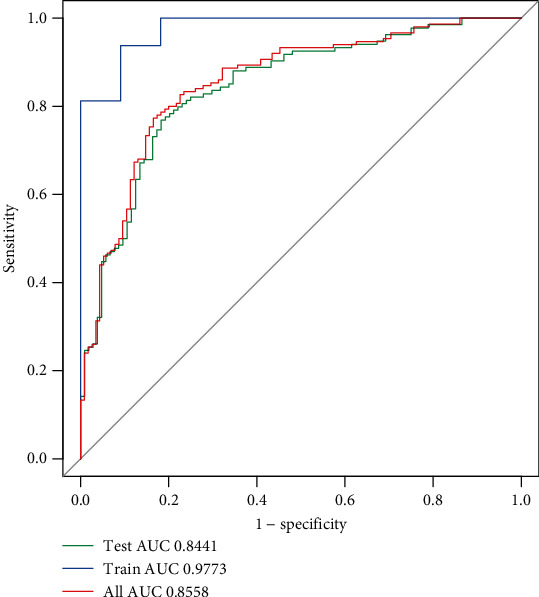
ROC analysis for comparison of the overall performance of the model using the different sets.

**Figure 7:**
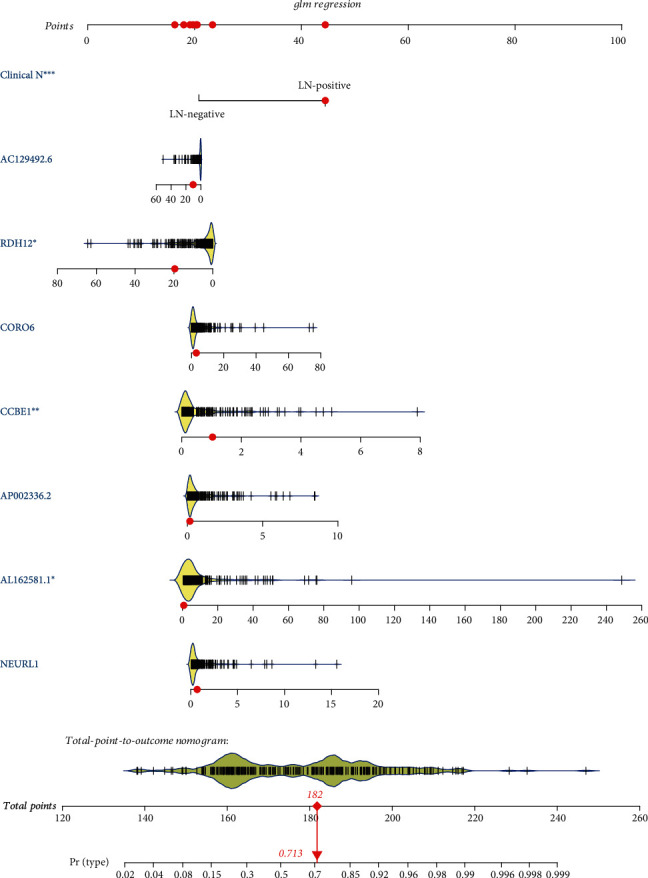
The nomogram for the prediction of lymph node metastasis.

Based on clinical N and seven candidate RNAs, we established a prediction model for LNM of OSCC. The machine learning was carried out, including LR, SVM, RF, and XGBoost. The sensitivity, specificity, positive predictive value (PPV), negative predictive value (NPV), accuracy area under the curve (AUC) mean value, and 95% confidence interval (CI) are shown in Table 2. From the result, the average of the accuracy rate of the machine learning model was 0.79 and the AUC value was 0.84, indicating that the model had optimal performance. Based on the machine learning model, the receiver operating curves (ROCs) were depicted in the training set, test set, and entire data (Figure 6). The AUCs were 0.9773, 0.8441, and 0.8558, respectively, suggesting the good prediction efficiency of the model. As shown in Figure 7, a nomogram was established to predict the risk for LNM in OSCC. Logistic regression analysis revealed that the clinical N stage (*p* < 0.001) was an important factor to predict LNM of OSCC. Furthermore, three DERNAs including RDH12 (*p* value < 0.05), CCBE1 (*p* value < 0.01), and AL162581.1 (*p* value < 0.05) possessed potential value as predictive biomarkers for LNM in OSCC patients.

**Figure 8:**
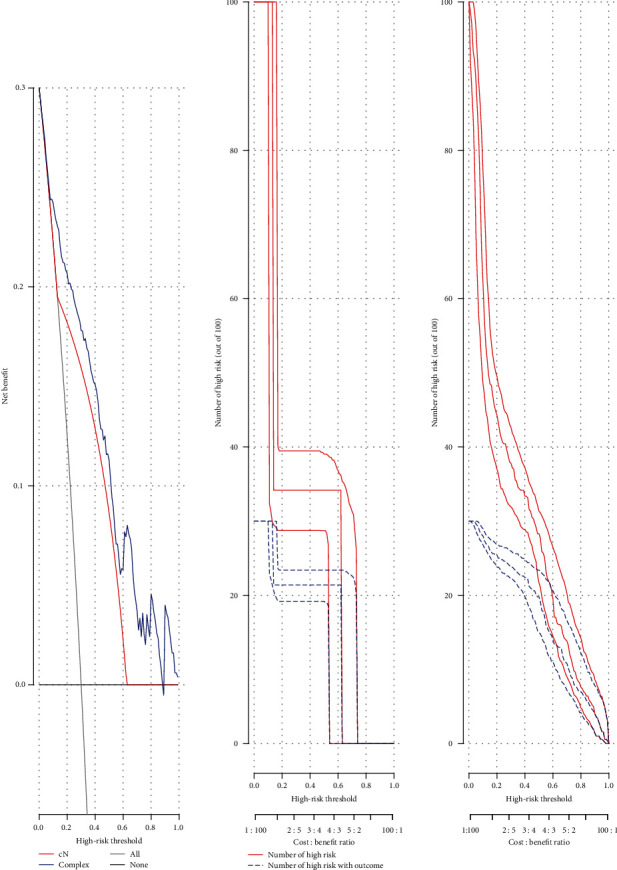
(a) The decision curve analyses (DCAs) for the clinical N stage and complex nomogram. (b) The clinical impact curve for the cN. (c) The clinical impact curve for the complex.

The following two logistic regression models were established, with cN as the predictor and pN as the outcome; the other was a multiple logistic regression model (complex), with cN and characteristic RNA expression levels as predictors and pN as the outcome. In Figure 8(a), a decision curve showed that using the RNA nomogram in the current study to predict the LNM added was more beneficial than only using the clinical N stage. As shown in Figures 8(b) and 8(c), our clinical impact curves draw the clinical influence curves of the cN model and the complex model, respectively. The simple model to predict the risk stratification of 100 patients displayed the “cost : benefit ratio” *y*-axis, assigned 8 scales, and showed the confidence interval. The red curve (number of high risk) represented the number of people who were classified as positive (high risk) by the cN model or the complex model at each threshold probability, and the blue curve (number of high risk with outcome) was the number of true positives at each threshold probability.

## 4. Discussion

LNM is the main reason for the failure of OSCC treatment, which will significantly reduce patients' survival rate [22, 23]. Once cervical LNM is detected during follow-up, cervical lymph node dissection does not always save the patient, and sometimes, the rescue rate is less than 40% [24]. At the same time, 60%-80% of END patients are evaluated as cN0 and no metastasis, but unnecessary cervical lymphadenectomy is required, which causes shoulder and neck pain and dysfunction, thereby affecting long-term quality of life (QOL) [25, 26]. Therefore, there is an urgent need to identify patients who can obtain greater benefits from END and to avoid unnecessary LN-related surgery in patients without LNM, especially in relatively early-stage T patients. The limitation of diagnostic imaging technology (including ultrasound, computed tomography (CT), and magnetic resonance imaging (MRI) is that LN status cannot be fully predicted. To date, many efforts have been made to develop diagnostic biomarkers for LNM in OCSS patients. However, most of these markers are limited by their detection potential.

Recent developments in technology of whole-transcriptome sequencing provide a possibility to develop new biomarkers and therapeutic strategies in most types of human cancers [8, 9]. In this study, we identified 139 DERNAs between non-LNM and LNM OSCC. These DEmRNAs were distinctly involved in several key pathways such as “regulation of striated muscle contraction,” “regulation of muscle system process,” and “muscle filament sliding,” indicating that these genes could be involved in LNM of OSCC. Based on DEGs, Sonohara et al. proposed a novel gene-expression signature for prediction of lymph node metastasis in esophageal squamous cell carcinoma (ESCC) patients by analyzing RNA-sequencing profiles [27]. Daisuke et al. constructed a 15-gene signature for detection of lymph node metastasis in early-stage gastric cancer (GC) patients by using the genome-wide transcriptomic approach [28]. With regard to OSCC, Pasini et al. developed a four-gene expression model to predict LNM in OSCC, but their results showed a 22% false positive rate in pN0 cases, which may lead to over treatment [29]. In the present study, we constructed an RNA-seq-based nomogram combined with clinicopathological factors. This model was composed of NEURL1, AL162581.1 (miscRNA), AP002336.2 (lncRNA), CCBE1, CORO6, RDH12, and AC129492.6 (pseudogene). These candidate DERNAs could significantly distinguish non-LNM and LNM of OSCC. Among them, CCBE1, CORO6, NEURL1, and RDH12 were significantly higher in N+ compared to N- OSCC patients in the GSE9844 dataset. More importantly, RDH12, CCBE1, and AL162581.1 were significantly associated with OSCC LNM. As per previous studies, CCBE1 is indispensable for the development of lymphatic vessels which have important roles in lymphangiogenesis and cancer metastasis [30]. Tumor lymphangiogenesis plays an important role in LNM of OSCC [31–33]. Hogan et al. suggested that CCBE1 may be an extracellular guidance molecule and an independent factor for normal lymphoblasts to germinate or even migrate [34]. Peng et al. concluded that CCBE1 had potential to be a biomarker for prediction of LNM in lung cancer patients because its expression was decreased in lung tumor tissue and further downregulated in patients with LNM [35]. Leong et al. found that CCBE1 as a direct target could promote invasion and metastasis of breast cancer [36]. Our research showed that analysis of CCBE1 expression in the OSCC tissues may help surgeons to evaluate the LNM risk, and CCBE1 might become a potentially therapeutic biomarker in OSCC. Further research is required to understand more about the function of CCBE1 in the LNM of OSCC.

Previous studies have shown that various clinicopathological predictors and genes are considered as risk factors for LNM in the patients with OSCC [37, 38]. However, no study has combined a visual presentation nomogram with these risk factors. The nomogram is a visualization of a statistical model specifically developed to optimize the accuracy of individual prediction. The preoperative nomogram of estimated LNM can help surgeons identify patients who can obtain greater benefit from a more extensive operation [39–41]. Compared with traditional multiple regression models, the advantage of the nomogram is that all key predictors are displayed graphically. Therefore, we established and validated a novel clinically useful nomogram based on RNA-seq combined with clinicopathological factors to predict the LNM of patients with OSCC. The AUC of the model was up to 0.9773 in the training set, suggesting that the model exhibited a good performance to predict LNM of OSCC. After validation by the test and entire sets, the model still possessed high sensitivity and accuracy on prediction of LNM of OSCC. Thus, the model established by comprehensive use of clinical features had good performance, and candidate RNAs were superior to the use of certain indicators alone, indicating the clinical practicality and innovation of our research. To further validate the prediction efficiency of the model on OSCC LNM, we conducted logistic regression models. As expected, the RNA nomogram combining the clinical N stage showed higher accuracy to predict LNM compared to only the clinical N stage.

At present, some studies have identified some individual markers for LNM in OCSS patients based on microarrays, which require separate clinical tests and individual clinical tests, resulting in increased costs [39–41]. The microarray technology used in those studies does not reflect current genomic views because it can only detect protein-coding genes. Combining protein-coding genes and noncoding genes may improve the robustness of molecular biomarkers. RNA-seq clinical tools have key advantages over other platforms. The bias and limitations of microarray data are improved by RNA-seq, especially in the detection of low-abundance transcripts. This advantage of RNA-seq can be translated into a better correlation with qPCR data in laboratory and patient samples, which is especially important for genes that tend to be differentially expressed but have low absolute abundance. Moreover, RNA-seq provides comprehensive expression data, which will become increasingly important in understanding and predicting the therapeutic response of most tumors that lack classic targetable changes. Our prediction model is based on the RNA-seq dataset. This method is more economical and more clinically applicable than whole-genome sequencing. Our findings constructed an RNA-seq-based nomogram combined with clinical pathology, which may provide clinicians with useful tools for preoperative prediction of LNM and tailor-made personalized treatment for OSCC patients. It is easy to understand its graphical scoring system, which helps to customize treatment and medical decisions. To the best of our knowledge, the RNA-seq-based nomogram described in this article has not been reported previously, providing a powerful prognostic tool for precision oncology. Therefore, this model may have important implications for clinical practice. Our RNA-seq data is based on the TCGA database and the study population is from different races, so the model can be extended to other races/ethnic populations. The RNA-seq-based nomogram combined with clinicopathology provides an opportunity for individualized adjuvant therapy based on biological factors and comprehensive change testing through the RNA-seq platform. Therefore, this model may be a clinically useful tool that can be easily incorporated into the RNA-seq clinical sequencing program to personalize OSCC treatment.

However, our study had certain limitations. We acknowledged that it was based on TCGA data and the sample size was relatively small. In addition, the nomogram had only been validated internally and still needed to be further validated by independent cohorts in a multicenter trial to investigate whether the results are applicable to other populations.

## 5. Conclusion

The RNA-seq-based nomogram combined with clinicopathology could potentially provide clinicians with a useful tool to best select patients with LNM who will benefit from neck dissection, while avoiding the cost and overtreatment of those without LNM. Ultimately, optimized individually tailored therapy will contribute to the management of OSCC patients based on the model.

## Figures and Tables

**Table 1:** The demographic characteristics of OSCC patients with or without lymph node metastasis.

Characteristics	OSCC without LNM (*n* = 119)	OSCC with LNM (*n* = 157)	*p* value
Gender			0.017^∗^
Male	74	119	
Female	45	38	
Age (years)	62.92 ± 12.91	59.89 ± 12.31	0.049^∗^
Clinical T			0.017^∗^
I-II	53	44	
III-IV	63	108	
Clinical N			<0.001^∗∗∗^
LN-negative	94	43	
LN-positive	21	107	
Neoplasm histologic grade			0.195
G1	23	17	
G2	77	104	
G3	18	31	
G4	0	1	
Tobacco smoking history			0.267
No	36	34	
Yes	81	120	
Margin status			0.148
Close	15	19	
Negative	90	108	
Positive	9	26	
Alcohol history			0.024^∗^
No	43	46	
Yes	70	110	
Anatomic neoplasm subdivision			0.5
Oral tongue	49	63	
Base of tongue	4	9	
Floor of mouth	23	33	
Buccal mucosa	8	12	
Alveolar ridge	11	5	
Oral cavity	21	32	
Hard palate	1	2	
Lip	2	1	

Note: ^∗^*p* value < 0.05, ^∗∗∗^*p* value < 0.001. OSCC: oral squamous cell carcinoma; LNM: lymph node metastasis.

**Table 2:** Comparison of the predictive performances of the machine learning model in the test set.

Model performances (HR, 95% CI)	RF	SVM	LR	XGBoost
Sensitivity	0.82 (0.75, 0.89)	0.80 (0.75, 0.85)	0.81 (0.74, 0.89)	0.72 (0.63, 0.81)
Specificity	0.67 (0.57, 0.78)	0.76 (0.68, 0.84)	0.76 (0.68, 0.84)	0.67 (0.57, 0.77)
PPV	0.77 (0.72, 0.83)	0.82 (0.77, 0.87)	0.82 (0.77, 0.88)	0.74 (0.67, 0.81)
NPV	0.76 (0.67, 0.84)	0.75 (0.71, 0.79)	0.78 (0.70, 0.83)	0.65 (0.56, 0.75)
Accuracy	0.75 (0.71, 0.80)	0.78 (0.75, 0.81)	0.79 (0.74, 0.83)	0.70 (0.62, 0.77)
AUC	0.82 (0.77, 0.88)	0.84 (0.80, 0.87)	0.84 (0.80, 0.89)	0.77 (0.71, 0.83)

RF: random forest; SVM: support vector machine; LR: logistic regression; HR: hazard ratio; CI: confidence interval; PPV: positive predictive value; NPV: negative predictive value; AUC: area under the curve.

## Data Availability

The datasets analyzed during the current study are available from the corresponding author on reasonable request.

## Abbreviations

LNM: Lymph node metastasis
OSCC: Oral squamous cell carcinoma
non-LNM: Without LNM
cN0: Clinically negative neck
END: Elective neck dissection
TCGA: The Cancer Genome Atlas database
GEO: Gene Expression Omnibus
OTSCC: Oral tongue squamous cell carcinoma
DERNAs: Differentially expressed RNAs
FC: Fold change
cN: Clinical N stage
SVM: Support vector machine
AUC: Area under the curve
LR: Logistic regression
RF: Random forest
N-: Pathological examination without lymph node metastasis
N+: Pathological examination with lymph node metastasis
HR: Hazard ratio
CI: Confidence interval
PPV: Positive predictive value
NPV: Negative predictive value.

## Supplementary Materials

Supplementary materials Supplementary Table 1: 139 differentially expressed mRNAs between non-LNM and LNM of OSCC.
